# Enteral nutritional strategy during therapeutic hypothermia: who? when? what?

**DOI:** 10.3389/fped.2024.1357831

**Published:** 2024-06-25

**Authors:** Simonetta Costa, Irene Del Rizzo, Simona Fattore, Francesca Serrao, Francesca Priolo, Mirta Corsello, Eloisa Tiberi, Milena Tana, Paola Catalano, Giovanni Vento

**Affiliations:** ^1^ Department of Woman and Child Health and Public Health, Catholic University of Sacred Heart, Fondazione Policlinico Universitario “A. Gemelli” IRCCS, Rome, Italy; ^2^ Department of Medicine, Surgery and Health Sciences, University of Trieste, Trieste, Italy

**Keywords:** birth asphyxia, enteral feeding, minimal enteral feeding, nutritional strategy, therapeutic hypothermia

## Abstract

**Background:**

There are no guidelines regarding enteral feeding (EF) of infants with hypoxic-ischemic encephalopathy (HIE) during and shortly after therapeutic hypothermia; consequently, clinical practice is, to date, still variable. The objective of this study is to assess whether a minimal EF strategy during therapeutic hypothermia may be associated with a shorter time to full EF of infants with HIE and to identify the clinical variables that independently affect the time to full EF.

**Methods:**

A retrospective study, covering the period from 1 January 2015 to 30 June 2022 was performed at the Neonatal Intensive Care Unit of the Fondazione Policlinico Universitario “Agostino Gemelli” IRCCS, Rome, which compared infants with HIE who received minimal EF during therapeutic hypothermia with those who did not.

**Results:**

Seventy-eight infants received minimal EF during therapeutic hypothermia, while 75 did not. Infants who were fed reached full EF significantly faster than those who were not. Moreover, they received parenteral nutrition and maintained central venous lines for a shorter time. A multivariate analysis, taking into account the variable of clinical severity, confirmed that minimal EF is an independent beneficial factor for reaching full EF in a shorter time and mechanical ventilation and seizures are independent factors for a longer time to full EF.

**Conclusions:**

Minimal EF during therapeutic hypothermia is associated with a shorter time to full EF in stable infants with HIE. Further prospective studies are needed to better define the enteral nutrition strategy for infants during therapeutic hypothermia, regardless of the severity of clinical conditions.

## 1 Introduction

Hypoxic-ischemic encephalopathy (HIE) represents a substantial cause of death and disability during the neonatal age (1). Currently, therapeutic hypothermia is the only validated strategy for reducing the long-term *sequelae* of HIE (2).

The optimal enteral feeding (EF) strategy for infants with HIE during therapeutic hypothermia is still debated (3). The studies available until now show the beneficial effect of EF without adverse effects, even if we remain uncertain about how to administer enteral nutrition to infants with HIE, and, above all, whether EF can be administered regardless of clinical severity (4–8).

A survey conducted in 2017 on the nutritional practices in neonatal intensive care units (NICUs) in the United Kingdom for infants treated with therapeutic hypothermia revealed that, of the 49 NICUs that answered, only 59% started EF during therapeutic hypothermia, with most initiating as a minimal EF (9).

The key reason for withholding EF during therapeutic hypothermia is the fear of necrotizing enterocolitis (NEC), which, although typically associated with prematurity, is described in full-term neonates with HIE (10).

Hypoxia is an established risk factor for NEC; hypoxia affects the gastrointestinal (GI) tract, decreasing perfusion and motility (11, 12). Given these assumptions, withholding EF might seem the most logical choice. However, from studies on very low birth weight (BW) infants, who are at greatest risk of NEC, it is known that the early provision of minimal EF is beneficial, as it stimulates GI motility, promotes GI hormone secretions, and favors beneficial microflora, thus improving GI tract maturation and feeding tolerance without increasing the risk of developing NEC (13). Moreover, therapeutic hypothermia seems to have a beneficial effect on GI function and feeding tolerance in moderate-to-severe HIE, as shown in studies comparing cooled infants with non-cooled ones (14–16).

Birth asphyxia typically affects visceral hemodynamics, with an increase in the superior mesenteric and celiac artery velocities in non-cooled infants with mild-to-moderate HIE during the first day of life and very low velocities in severe HIE. Therapeutic hypothermia plays a beneficial role by modulating the reperfusion phase and keeping blood flow stable during treatment, thus reducing the risk of reperfusion injury (17).

In this study, we aimed to assess whether a minimal EF strategy during therapeutic hypothermia may be associated with a shorter time to full EF in infants with HIE. In addition, we intended to identify the clinical variables that independently influence the achievement of full EF.

## 2 Methods

### 2.1 Settings and patients

We performed a monocentric retrospective cohort study at the NICU of the Fondazione Policlinico Universitario Agostino Gemelli IRCCS, Rome between 1 January 2015 and 30 June 2022.

We included all infants with HIE undergoing therapeutic hypothermia. Therapeutic hypothermia was initiated within 6 h of life in infants with a gestational age (GA) of ≥35 weeks and a BW of ≥1,800 g if the following two criteria were met: (1) intrapartum hypoxia with at least one of the following: (A) Apgar score ≤5 at 10 min, (B) persistent need for resuscitation at 10 min, and (C) blood gas acidosis characterized by either a pH of ≤7.0 or negative base excess of ≥12 mmol/L in the first hour of life; and (2) moderate to severe encephalopathy evaluated by standardized neurologic examination (18). Neurological involvement was confirmed by an amplitude-integrated electroencephalogram (aEEG), which was defined as moderately abnormal with an upper margin of >10 μV and a lower margin of <5 μV, severely abnormal with an upper margin of <10 μV and a lower margin of <5 μV, or burst suppression. Infants missing relevant nutritional and clinical data for the study were excluded.

Institutional review boards approved the study. Parents gave written informed consent to the processing of personal data.

### 2.2 Nutritional protocol during therapeutic hypothermia

In our NICU, the nutritional practice for infants with HIE undergoing therapeutic hypothermia involved the initiation of minimal EF if they did not have systemic hypotension requiring vasoactive therapy, disseminated intravascular coagulation, acidosis defined by a pH of <7.15 for more than 2 h, hypoxia defined by a PaO_2_ of <45 mmHg for more than 2 h, and abnormal abdominal clinical findings. Minimal EF was intended as trophic feeding with administration of 12–24 ml/kg/day of human or formula milk, delivered as an intermittent bolus by a nasogastric or an orogastric tube every 3 h. The total intravenous fluid volume was maintained between 50 and 60 ml/kg/day during therapeutic hypothermia.

Feeding was defined as well tolerated in the absence of pathological abdominal signs (distension, color changes, visible intestinal loops, or pain), pathological gastric residue (>100% of previous feeding, biliary, blood, or fecal content), vomiting, bloody stools, and associated cardiorespiratory events.

### 2.3 Primary and secondary outcomes

The primary outcome was the day of life at which a full EF of 150 ml/kg/day of milk was achieved and well tolerated for at least three consecutive days. Secondary outcomes included the duration of parenteral nutrition (PN) and central venous (CV) lines, breastfeeding rates at discharge, and length of hospital stay.

### 2.4 Data collection and definitions

For every newborn, we extracted data from the clinical records on sex, GA (determined by the best obstetric estimate based on the first day of the last menstrual period, prenatal ultrasound, and postnatal physical examination), BW, and small for GA (SGA) infants, defined with the Italian Neonatal Study (INeS) reference values as those with a BW *z*-score below −1.28 standard deviations (SDs) (19). Obstetrical diseases, the occurrence of sentinel events, outborn status, and mode of delivery were also recorded.

As for the severity of HIE, we detailed the Apgar score at 1, 5, and 10 min of life, the acid–base status at birth, the neurological examination before starting therapeutic hypothermia, the temperature on NICU admission, the aEEG pattern, the timing of the commencement of therapeutic hypothermia, and its target temperature.

We recorded data on the following neonatal morbidities: seizures; respiratory distress syndrome (RDS); pneumothorax; persistent pulmonary hypertension of the newborn (PPHN); transient tachypnea of the newborn; hypotension requiring inotropic support; acute kidney injury; anemia; thrombocytopenia and coagulation issues; pneumonia, defined by a positive bronchoalveolar lavage culture; late-onset culture-proven sepsis, defined by a positive blood culture occurring at or after 72 h of life; NEC according to Bell staging criteria (20); hypoglycemia, defined as a blood glucose concentration <47 mg/dl (21); cholestasis defined as the presence of serum-conjugated bilirubin with a level of >1 mg/dl when the total bilirubin was <5 mg/dl or a conjugated component of >20% of the total when the total bilirubin was >5 mg/dl (22).

We recorded data on neonatal treatments such as surfactant delivery, the percentage of infants on mechanical ventilation (MV) during therapeutic hypothermia, and the duration of MV, oxygen therapy, steroid treatment, inotropic drugs, diuretics, blood product transfusions, and vitamin K supplementation.

As for EF during therapeutic hypothermia, we registered the feeding volumes (ml/kg/day) for each day of therapeutic hypothermia, the type of milk (human milk or formula), feeding technique, and time to full EF.

Data on the duration of PN and CV lines, breastfeeding rates at discharge, and the length of neonatal unit stay were also registered.

### 2.5 Sample size and statistical analysis

We based the sample size calculation on our observational data, which showed that among the unfed infants undergoing therapeutic hypothermia, the mean ± SD time to reach the full EF was 10.0 ± 5.5 days. Sample size calculation demonstrated that at least 53 patients for each group were needed to detect a 3-day advantage in achieving full EF in fed infants with a power of 80% and an *α* error of 0.05 (23).

Data are reported as numbers (percentages) and mean ± SD for normally distributed variables and as median (interquartile range, IQR) for non-normally distributed variables.

We tested normal distribution using the Shapiro–Wilk test; we compared categorical variables with the chi-squared test or Fisher's exact test and continuous variables with the Student *t*-test for the independent sample or the Mann–Whitney *U* test.

We performed a univariate logistic regression analysis with minimal EF (stated as ml/kg/day on the third day of therapeutic hypothermia) as the independent variable and with time to full EF (stated as time to full EF of >9 days), CV line duration of >7 days, PN duration of >6 days, and sepsis as the dependent variables. We then performed a multivariate logistic regression analysis with minimal EF, MV, hypotension requiring inotropes, PPHN, severe HIE, and seizures as the independent variables and with time to full EF (stated as time to full EF of >9 days), CV line duration of >7 days, PN duration of >6 days, and sepsis as the dependent variables. A *p*-value <0.05 was considered statistically significant. We used software STATA 16 (StataCorp 2017).

## 3 Results

In total, 153 newborns met the inclusion criteria during the study period; among them, 78 infants received minimal EF during therapeutic hypothermia (fed infants), and 75 were not fed during therapeutic hypothermia (unfed infants).

Infants’ characteristics at baseline and obstetrical conditions are reported in Table 1. There were no significant differences in baseline characteristics and obstetrical conditions, except for a higher occurrence of sentinel events in the unfed infants and a higher occurrence of maternal hypertension among the fed infants. Unfed infants exhibited more severe initial clinical conditions, as evidenced by the lower Apgar scores at 1, 5, and 10 min and by the higher frequency of severe HIE according to the modified Sarnat staging system compared to the fed infants.

**Table 1:** Baseline characteristics between the two groups.

	Fed infants *N* = 78	Unfed infants *N* = 75	*p*-value
Gestational age, weeks	38.9 ± 1.5	38.8 ± 1.7	0.80
Late preterm	6 (8)	11 (15)	0.18
Term	72 (92)	64 (85)	0.18
Birthweight, g	3,290 ± 475	3,297 ± 566	0.93
AGA	59 (76)	56 (75)	0.89
SGA	11 (14)	9 (12)	0.70
LGA	8 (10)	10 (13)	0.55
Male, *n*	53 (68)	51 (68)	>0.99
Outborn infants, *n*	52 (67)	46 (61)	0.51
Maternal diabetes/hepatosis	18 (23)	17 (23)	>0.99
Chorioamnionitis	14 (18)	17 (23)	0.43
Maternal hypertension	18 (23)	4 (5)	0.02
Cesarean delivery	29 (37)	36 (48)	0.19
Sentinel events^a^	25 (32)	36 (48)	*0*.*047*
Apgar scores			
1 min	5 (5–6)	3 (1–5)	0.007
5 min	7 (6–8)	6 (5–7)	<0.001
10 min	8 (7–9)	7 (5–8)	0.008
pH at birth	7.04 ± 0.16	7.01 ± 0.19	0.36
Base excess at birth	−16.6 ± 5.5	−15.8 ± 5.5	0.69
Hypoxic-ischemic encephalopathy, *n*			
Moderate	73 (93)	55 (73)	<0.001
Severe	2 (3)	17 (23)	<0.001
Missing	3 (4)	3 (4)	>0.99
Body temperature at admission	35.1 ± 1.1	35 ± 1.3	0.67
Cerebral function monitoring, *n*			
Normal	10 (13)	6 (8)	0.43
Moderately abnormal	47 (60)	37 (49)	0.18
Severely abnormal	12 (15)	19 (25)	0.16
Burst suppression	2 (2.6)	6 (8)	0.16
Missing	7 (9)	7 (9)	>0.99
Starting timing of TH	4.5 ± 1.5	4.2 ± 1.3	0.22
Mean temperature during TH	33.5 ± 0.1	33.6 ± 0.2	0.15

TH, therapeutic hypothermia.

Data are shown as mean ± SD, median (interquartile range), and number (%).

^a^ Sentinel events were one of the following: umbilical cord accident (either cord prolapsed, knotted, torn, ruptured, or compressed), uterine rupture, placental abruption, shoulder dystocia, maternal hemorrhage or trauma, or cardiorespiratory arrest or seizures immediately preceding delivery.

Unfed infants were also sicker than fed infants, as evidenced by the higher rates of RDS requiring surfactant administration, PPHN, and hypotension requiring inotropic support (Table 2). The unfed cohort also showed significantly higher rates of MV during therapeutic hypothermia, late-onset culture-proven sepsis, and a longer duration of MV; they required corticosteroid and diuretic treatment more frequently, as well as platelet and fresh frozen plasma transfusions (Table 2).

**Table 2:** Morbidities and therapies between the two groups.

	Fed infants *N* = 78	Unfed infants *N* = 75	*p*-value
RDS needing surfactant therapy	5 (6)	18 (24)	0.003
PPHN	0	11 (15)	<0.001
Transient tachypnea of the newborn	5 (6)	5 (67)	>0.99
Pneumonia	2 (3)	6 (8)	0.16
Pneumothorax	3 (4)	3 (4)	>0.99
Late-onset, culture-positive blood sepsis	1 (1)	7 (9)	0.03
Necrotizing enterocolitis	0	0	>0.99
Acute renal failure	1 (1)	5 (6.7)	0.11
Seizures	17 (22)	24 (32)	0.2
Cholestasis	0	4 (4)	0.055
Mechanical ventilation, *n*	10 (13)	44 (59)	<0.001
Mechanical ventilation duration, days	0.35 ± 1.6	2.41 ± 2.5	<0.001
Oxygen therapy duration, days	1.4 ± 8.4	2.6 ± 4.3	0.26
Hypotension needing inotropic support	5 (6)	29 (38)	<0.001
Corticosteroids	1 (1)	7 (9)	0.032
Diuretics	1 (1)	8 (11)	0.016
Transfusions, *n*			
Red blood cells	7 (9)	15 (20)	0.07
Platelets	2 (3)	11 (15)	0.009
Fresh frozen plasma	8 (10)	23 (31)	0.002

Data are shown as mean ± SD or number (%).

Fed infants achieved full EF sooner, received fewer days of PN, and spent fewer days with a CV line compared to unfed infants. No statistically significant differences were found in the breastfeeding rates at the time of discharge or in the mean length of hospital stay between the groups (Table 3).

**Table 3:** Primary and secondary outcomes between the two groups.

	Fed infants *N* = 78	Unfed infants *N* = 75	*p*-value
Time to FEF, days	7.6 ± 3.5	9.8 ± 5.5	0.004
PN duration, days	4.8 ± 3.6	7.2 ± 6.3	0.004
Central venous line duration, days	6 ± 3.7	9.1 ± 8.2	0.002
Breastfeeding at discharge, *n*	61 (78)	60 (80)	>0.99
Length of hospital stay, days	15.9 ± 20.6	21.1 ± 28.8	0.20

Data are shown as mean ± SD and number (%).

Regarding the fed infants, Table 4 presents the day of therapeutic hypothermia at which minimal EF started, the volume of feedings, the type of milk, and the feeding technique. Most patients started minimal EF on the third day of therapeutic hypothermia. Only a minority of patients received minimal EF throughout the entire therapeutic hypothermia course. All patients received a true minimal EF on day 3 of therapeutic hypothermia. The most common feeding technique was bolus tube feeding, but a large number of infants were also bottle-fed. Only a minority of infants received their mother's milk.

**Table 4:** Minimal enteral feeding during TH.

Feeding volume (ml/kg/day)	
Day 1	0.4 ± 1.9
Day 2	3.5 ± 5.8
Day 3	12.9 ± 6.9
When (day of TH)	
Day 1	5 (6)
Day 2	22 (28)
Day 3	51 (65)
How (mode of feeding)	
Bottle, *n*	30 (38)
Bolus tube feeding, *n*	42 (54)
Continuous tube feeding, *n*	6 (8)
What (type of milk)	
Human milk	14 (18)
Formula	64 (82)

FEF, full enteral feeding.

Data are shown as mean ± SD and number (%).

We then performed a logistic regression analysis to examine whether minimal EF was associated with a shorter time to full EF and with other clinical outcomes. In the univariate regression analysis, minimal EF emerged as a beneficial factor for achieving time to full EF of less than 9 days, as well as for CV line duration of less than 7 days, PN duration of less than 6 days, and for preventing late-onset sepsis (Table 5). In the multivariate regression analysis, we included the variable of clinical severity (the need for mechanical ventilation seizures, hypotension requiring inotropes, PPHN, and severe HIE). Minimal EF was confirmed as an independent beneficial factor for a time to full EF of less than 9 days, whereas MV and the presence of clinical seizures were independent variables associated with a longer time to full EF (Table 5).

**Table 5:** Logistic regression analysis.

	OR	95% CI	*p*
Univariate logistic regression for MEF on clinical outcomes			* *
Time to FEF > 9 days	0.185	0.08–0.426	<0.001
Central venous line duration > 7 days	0.46	0.054–0.326	<0.001
Parenteral nutrition > 6 days	0.156	0.063–0.387	<0.001
Late-onset, culture-proven sepsis	0.095	0.012–0.772	0.028
Multivariate logistic regression results for time to FEF > 9 days			
MEF	0.331	0.113–0.970	0.044
Mechanical ventilation	1.429	1.120–1.822	0.004
Seizures	3.433	1.288–9.155	0.014
Hypotension requiring inotropes	1.684	0.555–5.112	0.357
PPHN	0.645	0.131–3.174	0.589
Severe HIE	0.413	0.106–1.611	0.203
Constant	0.210	—	<0.001

MEF, minimal enteral feeding; FEF, full enteral feeding.

## 4 Discussion

In this monocentric retrospective study, we found that feeding infants with minimal EF during therapeutic hypothermia was associated with a significant reduction of the time to full EF and a reduction of the PN and CV line duration.

Moreover, we identified minimal EF as a beneficial independent factor for a time to full EF of less than 9 days, whereas MV and the presence of clinical seizures during therapeutic hypothermia were identified as independent variables associated with a longer time to full EF.

The available reports on the effects of minimal EF on the time to full EF are conflicting. A retrospective study comparing 34 cooled infants cared for in the United Kingdom, where feeds are commonly withheld, with 51 infants cared for in Sweden, where feeding was commonly administered during therapeutic hypothermia, failed to find a difference in the time to full EF between the two groups (4).

Our findings are instead in agreement with those of another small, retrospective, matched case–control study, which compared 17 cooled infants who received minimal EF with 17 infants who did not; in that study, minimal EF during hypothermia was associated with a reduced length of stay and a shorter time to full EF (5).

Our results are also in agreement with a recent randomized controlled trial aiming to explore the effect of early (during therapeutic hypothermia) vs. delayed (after therapeutic hypothermia) enteral nutrition on the incidence of feeding intolerance among cooled infants (8). Although the study found a similar incidence of feeding intolerance between the two study groups, the time to full EF was shorter in cooled infants who received EF during therapeutic hypothermia compared with those who received enteral nutrition after rewarming. In that study, clinical variables that could have influenced the time to full EF, such as the percentage of infants on MV during therapeutic hypothermia, the duration of MV, or the presence of seizures, were not declared.

At last, a large retrospective cohort study involving 6,030 newborns with HIE assessed the effect of EF during therapeutic hypothermia on the rates of NEC, finding that the incidence of NEC was lower in cooled infants who were fed (6). There was no mention of the time to full EF, although the duration of PN, which can be considered a surrogate for the earlier achievement of full EF, was shorter in the fed infants (6). In the same study, some clinical variables suggestive of sickness that are discernible to clinicians and could influence decision making regarding enteral nutrition, such as MV during therapeutic hypothermia or the presence of seizures, were not reported; however, above all, despite the great methodological soundness of the study, the criteria for identifying the most appropriate infants with HIE to start feeding early were not established.

In our cohort of cooled infants, 65% of infants started minimal EF on the third day of therapeutic hypothermia and only 6% of infants received minimal EF throughout the entire therapeutic hypothermia course. The starting time of enteral nutrition was not reported in all of the previous papers, but it appears that enteral nutrition was started on average on the second day of therapeutic hypothermia (5, 8). In our study, all infants received a true minimal EF (an amount of milk ranging from 12 to 24 ml/kg/day) on the third day of therapeutic hypothermia. These data appear to be comparable to that reported by other studies (4, 5, 8).

Therefore, the debate about enteral nutrition strategy during therapeutic hypothermia is still ongoing, and several questions still need to be addressed: Should all asphyxiated infants receive EF during the course of therapeutic hypothermia, regardless of the severity of the illness? What is the optimal day for initiating EF? What should be the optimal feeding volume?

Randomized controlled trials are warranted to develop an efficient feeding protocol for asphyxiated infants undergoing therapeutic hypothermia.

The major limitation of our study is its retrospective nature, which did not allow us to have two cohorts of infants with comparable clinical features of severity and to exclude other variables, in addition to those considered, that could have had an effect on the time to full EF. Another limitation of the study is that only 6% of the fed infants received minimal EF throughout the entire therapeutic hypothermia course, while 65% began minimal EF on the third day of therapeutic hypothermia, leading to enteral nutritional disparity within the group. This may reflect clinicians’ fear of feeding cooled infants even when clinical conditions do not contraindicate starting minimal EF.

Our study also has many strengths: the study was well equipped to detect differences in the primary outcome; furthermore, many variables indicative of illness severity were measured, and, most importantly, a statistical analysis was conducted to identify the clinical variables potentially associated with time to full EF.

## 5 Conclusions

The introduction of minimal EF during therapeutic hypothermia is associated with a shorter time to full EF, a shorter duration of PN and CV lines, and a lower incidence of late-onset sepsis. Randomized controlled trials are still necessary to identify the optimal enteral nutrition strategy for cooled infants to understand whether they could be fed regardless of illness severity, to determine when enteral nutrition should be started during therapeutic hypothermia, and to know how cooled infants should be fed, including considerations such as the quantity and quality of milk.

## Data Availability

The raw data supporting the conclusions of this article will be made available by the authors without undue reservation.
